# Supplementary material for: Group Cognitive Behavioural Therapy for Non-Rapid Eye Movement Parasomnias: Long-Term Outcomes and Impact of COVID-19 Lockdown
Source: Brain Sci. 2023 Feb 17;13(2):347. doi: 10.3390/brainsci13020347 (PMC9953888; doi:10.3390/brainsci13020347)
Supplement: Supplementary file 1 [file brainsci-13-00347-s001.zip › brainsci-2130329-supplementary.pdf]

# Supplement to: Group Cognitive Behavioural Therapy for Non-Rapid Eye Movement Parasomnias: Long-term Outcomes and Impact of COVID-19 Lockdown

## Supplement Outline


1. **Methods and Protocol**
2. **ICL Questionnaire**
3. **Supplementary Results**


## 1. *Methods and Protocol*

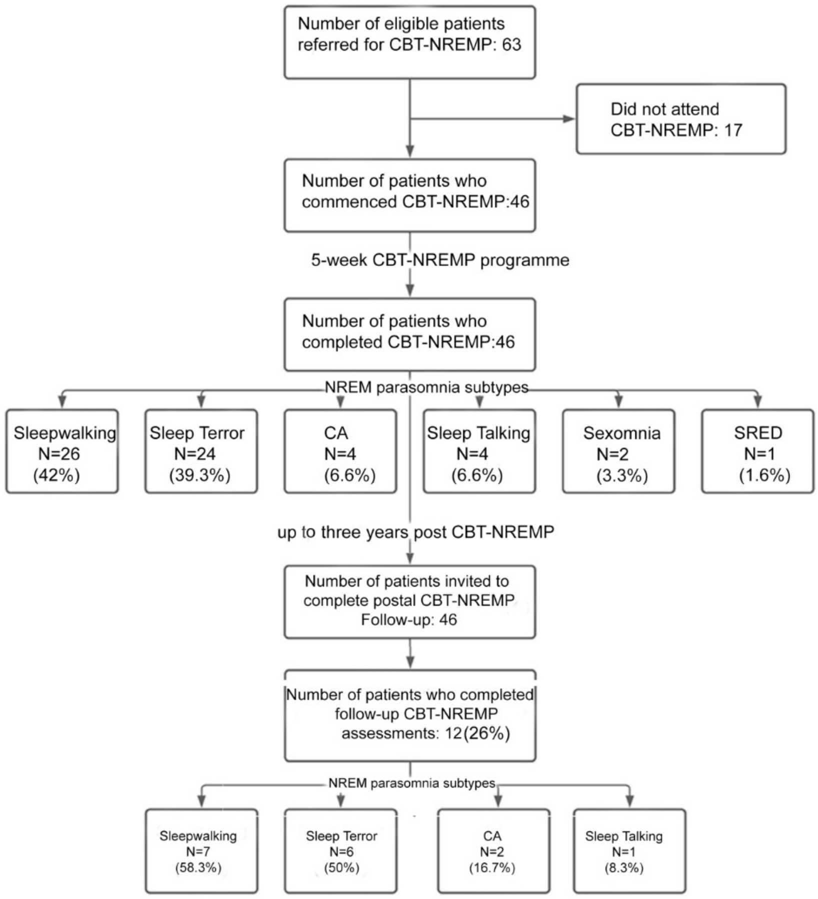


**Figure S1.** Flowchart of the studied cohort of patients with NREM parasomnia. Some patients reported N>1 subtype of NREM parasomnia. Percentages indicate the prevalence of each NREM parasomnia subtype in our cohort. CBT-NREMP: Cognitive Behavioural Therapy for NREM parasomnia; CA: confusional arousal; SRED: sleep-related eating disorder; NREM: non-REM; N: number.

## 2. Impact of COVID-19 Lockdown on NREM Parasomnias Questionnaire (ICL)

### Questionnaire S1. IMPACT of COVID-19 lockdown on NREM Parasomnias Questionnaire

*The purpose of this questionnaire is to understand if COVID-19 lockdown impacted your NREM parasomnias in any way. Please complete the questionnaire based on your overall experience of the COVID-19 lockdown to date. If a question is non-applicable to you, please circle the 'no change' option, or write N/A in the margin.*

---

***Starting Questions - please circle the best option for you***

Did you contract Covid-19?: yes/no

If yes, did you require hospital treatment?: yes/no

Were you home confined during lockdown (i.e. Did you work from home; remain essentially housebound)?: yes/no

Were you classified as an essential worker during lockdown (e.g. healthcare, police, fire service, or in a job that is COVID-19 essential?): yes/no

Did you live alone during lockdown? yes/no

---

***Sleep***

My NREM parasomnias deteriorated during lockdown (e.g. my sleepwalking became more frequent)

| Strongly Disagree | | | | No Change | | | | | Strongly Agree |
|---|---|---|---|---|---|---|---|---|---|
| 1 | 2 | 3 | 4 | 5 | 6 | 7 | 8 | 9 | 10 |

My sleep overall deteriorated during lockdown

| Strongly Disagree | | | | No Change | | | | | Strongly Agree |
|---|---|---|---|---|---|---|---|---|---|
| 1 | 2 | 3 | 4 | 5 | 6 | 7 | 8 | 9 | 10 |

Lockdown continues to adversely affect my sleep

| Strongly Disagree | | | | No Change | | | | | Strongly Agree |
|---|---|---|---|---|---|---|---|---|---|
| 1 | 2 | 3 | 4 | 5 | 6 | 7 | 8 | 9 | 10 |

During lockdown, …

A. It took me longer to fall asleep

| Strongly Disagree | | | | No Change | | | | | Strongly Agree |
|---|---|---|---|---|---|---|---|---|---|
| 1 | 2 | 3 | 4 | 5 | 6 | 7 | 8 | 9 | 10 |

B. I was awake for more of the night

| Strongly Disagree | | | | No Change | | | | | Strongly Agree |
|---|---|---|---|---|---|---|---|---|---|
| 1 | 2 | 3 | 4 | 5 | 6 | 7 | 8 | 9 | 10 |

C. I found it difficult to get up on time

| Strongly Disagree | | | | No Change | | | | | Strongly Agree |
|---|---|---|---|---|---|---|---|---|---|
| 1 | 2 | 3 | 4 | 5 | 6 | 7 | 8 | 9 | 10 |

D. I napped more during the day

| Strongly Disagree | | | | No Change | | | | | Strongly Agree |
|---|---|---|---|---|---|---|---|---|---|
| 1 | 2 | 3 | 4 | 5 | 6 | 7 | 8 | 9 | 10 |

E. My sleep quality overall was poorer

| Strongly Disagree | | | | No Change | | | | | Strongly Agree |
|---|---|---|---|---|---|---|---|---|---|
| 1 | 2 | 3 | 4 | 5 | 6 | 7 | 8 | 9 | 10 |

F. I felt more tired and fatigued during the day

| Strongly Disagree | | | | No Change | | | | | Strongly Agree |
|---|---|---|---|---|---|---|---|---|---|
| 1 | 2 | 3 | 4 | 5 | 6 | 7 | 8 | 9 | 10 |

G. It was more difficult to switch off my mind at night

| Strongly Disagree | | | | No Change | | | | | Strongly Agree |
|---|---|---|---|---|---|---|---|---|---|
| 1 | 2 | 3 | 4 | 5 | 6 | 7 | 8 | 9 | 10 |

---

*Mental Health*

I feel that my overall mental health deteriorated during lockdown

| Strongly Disagree | | | | No Change | | | | | Strongly Agree |
|---|---|---|---|---|---|---|---|---|---|
| 1 | 2 | 3 | 4 | 5 | 6 | 7 | 8 | 9 | 10 |

I felt anxious during lockdown

| Strongly Disagree | | | | No Change | | | | | Strongly Agree |
|---|---|---|---|---|---|---|---|---|---|
| 1 | 2 | 3 | 4 | 5 | 6 | 7 | 8 | 9 | 10 |

I felt depressed during lockdown

| Strongly Disagree | | | | No Change | | | | | Strongly Agree |
|---|---|---|---|---|---|---|---|---|---|
| 1 | 2 | 3 | 4 | 5 | 6 | 7 | 8 | 9 | 10 |

I felt more stressed during lockdown

| Strongly Disagree | | | | No Change | | | | | Strongly Agree |
|---|---|---|---|---|---|---|---|---|---|
| 1 | 2 | 3 | 4 | 5 | 6 | 7 | 8 | 9 | 10 |

---

***General wellbeing and lifestyle***

My ability to engage in exercise during lockdown reduced

| Strongly Disagree | | | | No Change | | | | | Strongly Agree |
|---|---|---|---|---|---|---|---|---|---|
| 1 | 2 | 3 | 4 | 5 | 6 | 7 | 8 | 9 | 10 |

My alcohol consumption increased during lockdown

| Strongly Disagree | | | | No Change | | | | | Strongly Agree |
|---|---|---|---|---|---|---|---|---|---|
| 1 | 2 | 3 | 4 | 5 | 6 | 7 | 8 | 9 | 10 |

My use of illicit substances (e.g. cannabis, cocaine, LSD etc) increased during lockdown

| Strongly Disagree | | | | No Change | | | | | Strongly Agree |
|---|---|---|---|---|---|---|---|---|---|
| 1 | 2 | 3 | 4 | 5 | 6 | 7 | 8 | 9 | 10 |

My diet deteriorated during lockdown (e.g. I gained weight and/or ate more unhealthily)

| Strongly Disagree | | | | No Change | | | | | Strongly Agree |
|---|---|---|---|---|---|---|---|---|---|
| 1 | 2 | 3 | 4 | 5 | 6 | 7 | 8 | 9 | 10 |

I felt lonely during lockdown

| Strongly Disagree | | | | No Change | | | | | Strongly Agree |
|---|---|---|---|---|---|---|---|---|---|
| 1 | 2 | 3 | 4 | 5 | 6 | 7 | 8 | 9 | 10 |

Lockdown affected my family and social relationships

| Strongly Disagree | | | | No Change | | | | | Strongly Agree |
|---|---|---|---|---|---|---|---|---|---|
| 1 | 2 | 3 | 4 | 5 | 6 | 7 | 8 | 9 | 10 |

My ability to connect and communicate with others deteriorated during lockdown

| Strongly Disagree | | | | No Change | | | | | Strongly Agree |
|---|---|---|---|---|---|---|---|---|---|
| 1 | 2 | 3 | 4 | 5 | 6 | 7 | 8 | 9 | 10 |

I have been adversely affected financially due to lockdown

| Strongly Disagree | | | | No Change | | | | | Strongly Agree |
|---|---|---|---|---|---|---|---|---|---|
| 1 | 2 | 3 | 4 | 5 | 6 | 7 | 8 | 9 | 10 |

Working from home adversely affected my general wellbeing

| Strongly Disagree | | | | No Change | | | | | Strongly Agree |
|---|---|---|---|---|---|---|---|---|---|
| 1 | 2 | 3 | 4 | 5 | 6 | 7 | 8 | 9 | 10 |

My responsibilities increased during lockdown e.g. I had to homeschool my children

| Strongly Disagree | | | | No Change | | | | | Strongly Agree |
|---|---|---|---|---|---|---|---|---|---|
| 1 | 2 | 3 | 4 | 5 | 6 | 7 | 8 | 9 | 10 |

The ill effects of lockdown are still impacting my general wellbeing

| Strongly Disagree | | | | No Change | | | | | Strongly Agree |
|---|---|---|---|---|---|---|---|---|---|
| 1 | 2 | 3 | 4 | 5 | 6 | 7 | 8 | 9 | 10 |

---

### *3. Supplementary Results*

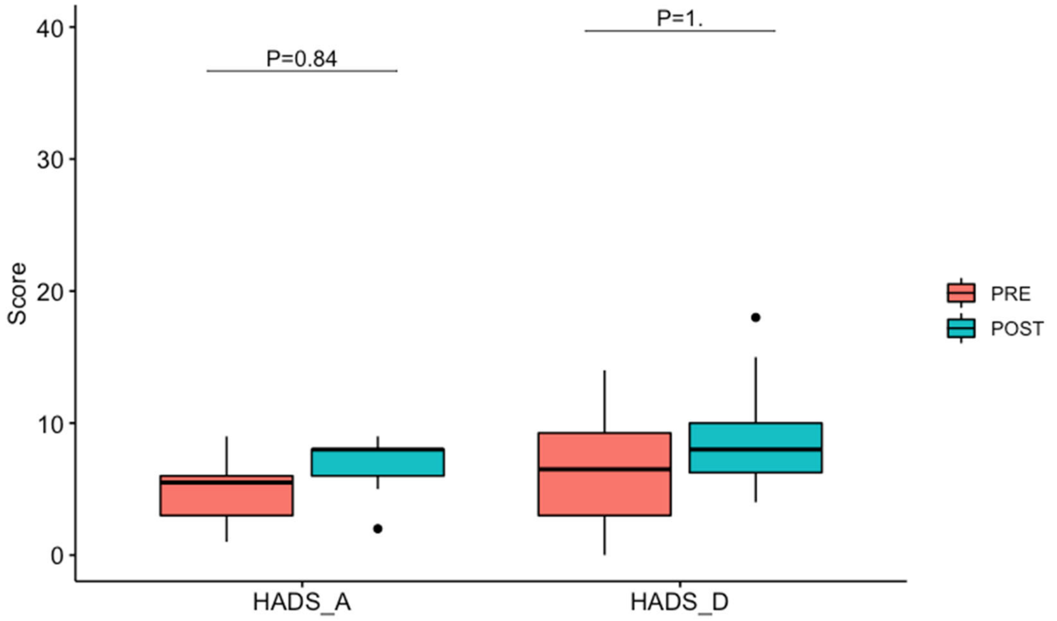


**Figure S2**. Box plot of Assessment Score for PRE (after the CBT-NREMP intervention) and during lockdown (POST) for HADS components (note: P values are from Wilcoxon tests). PRE: post CBT-NREMP treatment; POST: follow-up. Box represents the 50% of the central data (between 25$^{th}$ and 75$^{th}$ percentiles), with a line inside that

represents the median. Dots represent points outside 1.5 times the interquartile range above the upper quartile and below the lower quartile.

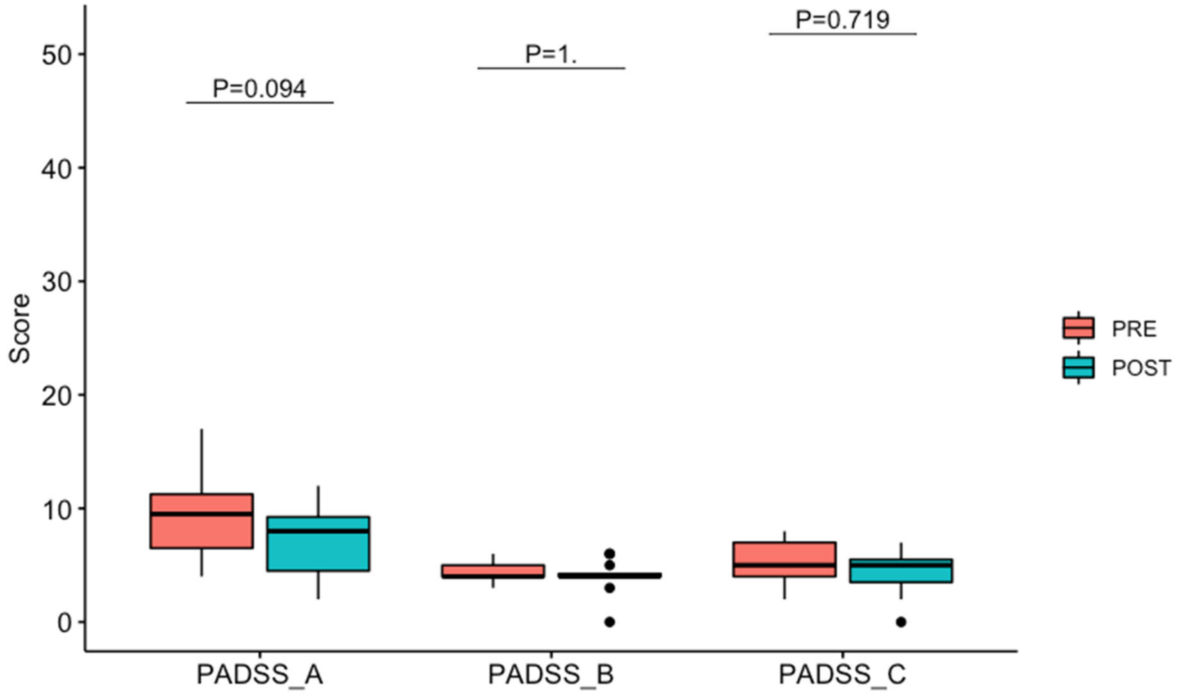


**Figure S3.** Box plot of Assessment Score for PRE and POST for PADSS components (note: p values are from Wilcoxon tests). PRE: post CBT-NREMP treatment; POST: follow-up. Box represents the 50% of the central data (between 25th and 75th percentiles), with a line inside that represents the median. Dots represent points outside 1.5 times the interquartile range above the upper quartile and below the lower quartile.

**Table S1.** Pairwise comparison of pre-treatment, post-treatment and the current follow-up (lockdown) time points for scales of ISI, HADS, and PADSS using Wilcoxon signed rank test along with the adjusted p values for multiple tests using Bonferroni correction.

| | | Wilcoxon signed rank test statistics | P value | Adjusted p value |
|---|---|---|---|---|
| **Pre- (before CBT-NREMP intervention) vs Post- (after CBT-NREMP intervention) treatment comparison** | | | | |
| ISI | ISI | 42.5 | 0.137 | 1 |
| HADS | HADS | 49.5 | 0.432 | 1 |
| HADS_A | HADS_A | 37 | 0.353 | 1 |
| HADS_D | HADS_D | 41.5 | 0.475 | 1 |
| PADSS | PADSS | 38.5 | 0.281 | 1 |
| PADSS_A | PADSS_A | 33.5 | 0.207 | 1 |
| PADSS_B | PADSS_B | 1.5 | 1 | 1 |
| PADSS_C | PADSS_C | 18.5 | 0.667 | 1 |
| **Pre-(before CBT-NREMP intervention) vs Current F/U (postal survey) comparison** | | | | |
| ISI | ISI | 51 | 0.365 | 1 |
| HADS | HADS | 17.5 | 0.332 | 1 |
| HADS_A | HADS_A | 1.5 | 0.073 | 1 |
| HADS_D | HADS_D | 22.5 | 0.646 | 1 |
| PADSS | PADSS | 52.5 | 0.012* | 0.295 |
| PADSS_A | PADSS_A | 65 | 0.005* | 0.119 |
| PADSS_B | PADSS_B | 3 | 0.371 | 1 |
| PADSS_C | PADSS_C | 26 | 0.049 | 1 |
| **Post-(after CBT-NREMP intervention) vs Current F/U (postal survey) comparison** | | | | |
| ISI | ISI | 42 | 0.844 | 1 |
| HADS | HADS | 11 | 0.102 | 1 |
| HADS_A | HADS_A | 6 | 0.105 | 1 |
| HADS_D | HADS_D | 12 | 0.438 | 1 |
| PADSS | PADSS | 50 | 0.024* | 0.583 |
| PADSS_A | PADSS_A | 52.5 | 0.012* | 0.283 |
| PADSS_B | PADSS_B | 6 | 0.174 | 1 |
| PADSS_C | PADSS_C | 19 | 0.09 | 1 |

Abbreviations: CBT-NREMP, Cognitive Behavioural Therapy for Non-REM parasomnia; F/U, follow-up (i.e. the current postal survey); ISI, Insomnia Severity Index; PADSS, Paris Arousal Disorders Severity Scale (total score); PADSS-A, Paris Arousal Disorders Severity Scale-subset A score; PADSS-B; Paris Arousal Disorders Severity Scale subset-B score; PADSS-C, Paris Arousal Disorders Severity Scale subset-C score; HADS, Hospital Anxiety and Depression Scale (total score); HADS-A, Hospital Anxiety and Depression Scale-Anxiety subset score; HAD-D, Hospital Anxiety and Depression Scale - Depression subset score. Statistically significant values are shown. *Wilcoxon signed rank test, along with adjusted p values for multiple tests using Bonferroni correction.

**Table S2.** Impact of Covid-19 Lockdown on depression, anxiety and each of the sleep-related scores (as measured via the ICLQ), using one sample Wilcoxon signed-rank test.

| Variables | Z -Score | P-Value $< 0.05$ |
|---|---|---|
| **NREM parasomnia deteriorated** | **2.274** | **0.023** |
| Sleep deteriorated | 1.791 | 0.073 |
| Lockdown continues to adversely affect my sleep | 0.060 | 0.952 |
| Increased sleep onset latency | -0.085 | 0.932 |
| Increased wake-after-sleep-onset | 0.000 | 1.000 |
| **Difficult to rise on time** | **2.506** | **0.012** |
| Increased excessive daytime somnolence | -0.120 | 0.905 |
| Poor sleep quality | 1.521 | 0.128 |
| **Increased daytime tiredness** | **2.039** | **0.041** |
| **Overactive mind at night** | **2.620** | **0.009** |
| Mental health deteriorated | 1.655 | 0.098 |
| **Increased anxiety** | 2.210 | **0.027** |
| Increased depression | 1.299 | 0.194 |
| **Increased stress** | **2.032** | **0.042** |
| Reduced engagement with exercise | 0.861 | 0.389 |
| Increased alcohol consumption | 1.590 | 0.112 |
| **Increased illicit substance use** | **-2.222** | **0.026** |
| **My diet deteriorated** | **2.070** | **0.038** |
| Increased loneliness | 0.568 | 0.570 |
| Relationships were adversely affected | 1.206 | 0.228 |
| Ability to communicate with others were adversely affected | 1.206 | 0.228 |
| Finances were adversely affected | 0.060 | 0.952 |
| Working from home had a negative impact on my general wellbeing | 1.478 | 0.139 |
| My responsibilities increased | -0.085 | 0.932 |
| The negative effects of lockdown continue to impact my general wellbeing | 0.981 | 0.327 |

Abbreviations: ICLQ = Impact of COVID-19 Lockdown Questionnaire.

A one-sample Wilcoxon signed-rank test revealed significant effects across eight items. The main effects were primarily observed in the questionnaire's sleep subscale, where lockdown measures were shown to contribute to: the deterioration of NREM parasomnia (*P* = .023; M = 6.5; Table 4, Figure 3), patients' ability to wake up on time and sleep inertia (*P* = .012; M = 7), additional tiredness during the day or excessive daytime somnolence (*P* = .041; M = 7) and difficulties switching the mind off at night or hypervigilance (*P* = .009; M = 7.5; Table 4, Figure 4). Moreover, congruent with these scores, in the mental health, general wellbeing and lifestyle subscales, patients also reported: elevated levels of anxiety during lockdown (*P* = .027; M: 7; Tables 3, 4, Figure 4), increased amount of stress (*P* = .042 M: 8), dietary deterioration (*P* = .038; M: 7), but lower levels of illicit substance use (*P* = .026; M: 4.5). *Statistically significant results are indicated in bold*

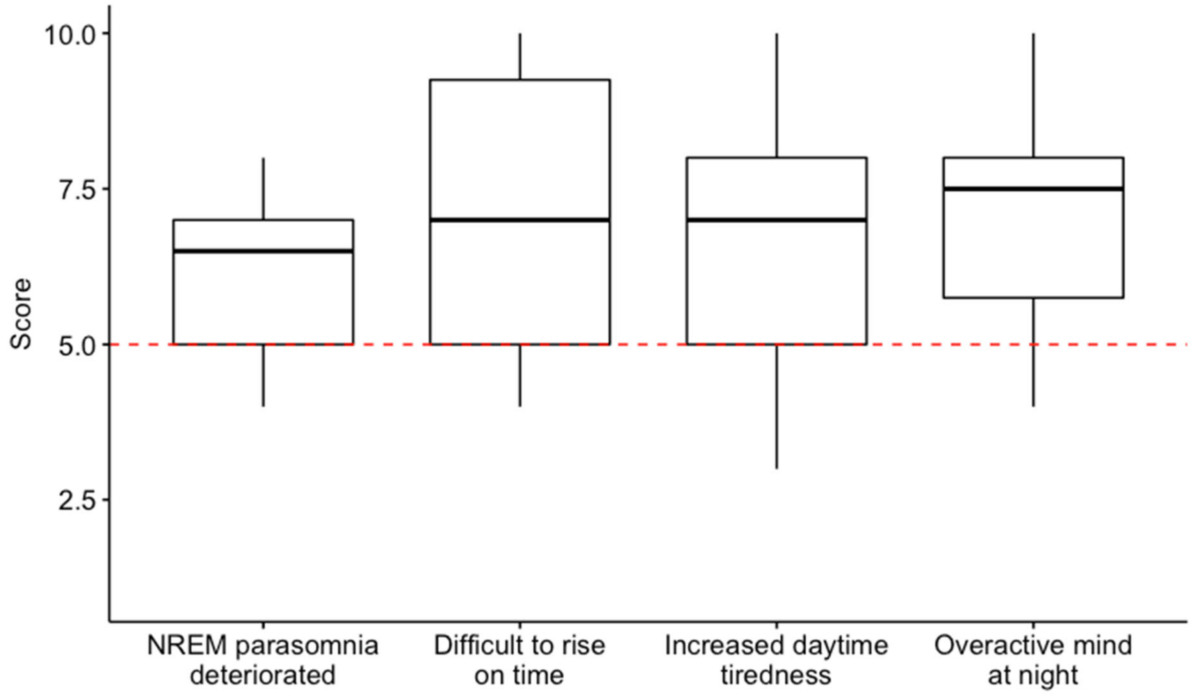


**Figure S4.** Box plot of significant effects observed in sleep subscales. *Red dotted line indicates a score of five which indicates no change in effect.* Box represents the 50% of the central data (between 25th and 75th percentiles), with a line inside that represents the median. Dots represents points outside 1.5 times the interquartile range above the upper quartile and bellow the lower quartile.

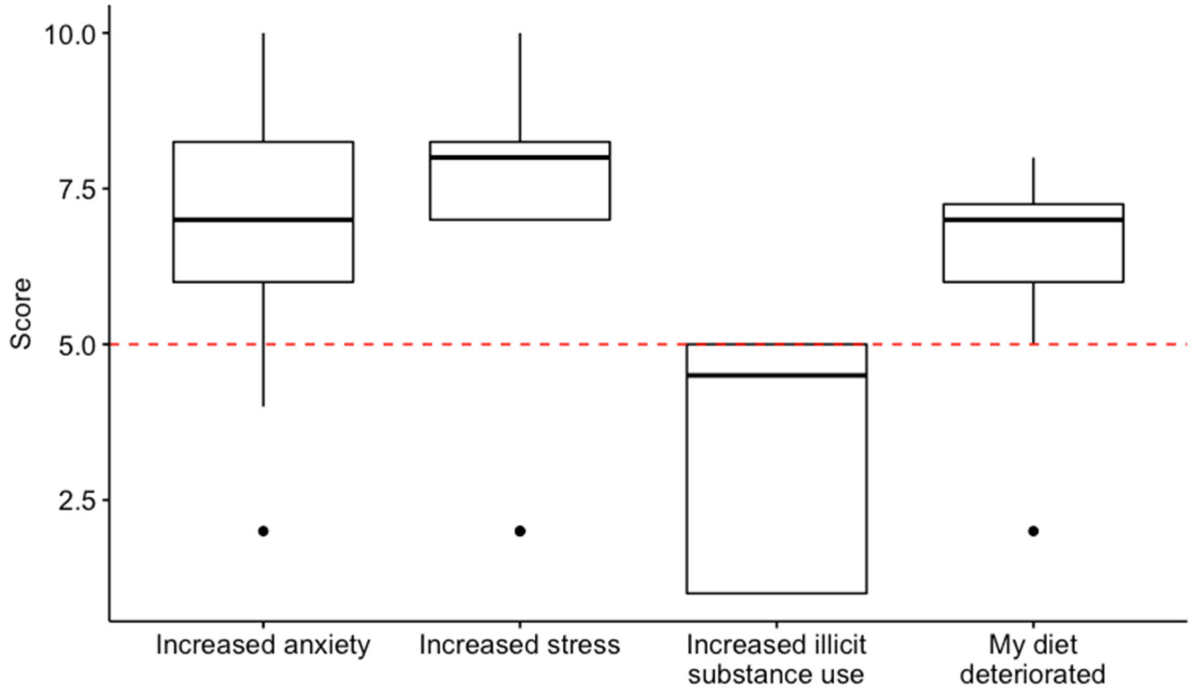


**Figure S5.** Box plot of significant effects observed in mental health, general wellbeing and lifestyle subscales. *Red dotted line indicates a score of five which indicates no change in effect.* Box represents the 50% of the central data (between 25th and 75th percentiles), with a line inside that represents the median. Dots represents points outside 1.5 times the interquartile range above the upper quartile and bellow the lower quartile.
